# “We Could Hold Our Own Here at Home”: Longitudinal Experience of COVID‐19 Lockdowns in Parents With Children Affected With Interstitial Lung Disease

**DOI:** 10.1002/ppul.27446

**Published:** 2024-12-18

**Authors:** Carlee Gilbert, Andrew Bush, Kate M. Bennett, Christopher Brown

**Affiliations:** ^1^ Institute for Population Health University of Liverpool England UK; ^2^ Imperial College, National Heart and Lung Institute; Royal Brompton Harefield NHS Foundation Trust London UK

**Keywords:** adaptation, childhood interstitial lung disease, COVID‐19, lived experience, parents, qualitative, resilience, uncertainty, young people

## Abstract

The global health emergency of COVID‐19 in early 2020 placed much of the population under quarantine. Interstitial Lung Disease in childhood (chILD) was recommended to be a pediatric clinically extremely vulnerable (CEV) group in April 2020 for shielding due to the unknown health consequences of COVID‐19 in children with chronic respiratory conditions. This qualitative longitudinal research study explores how chILD parents in the UK experienced COVID‐19 lockdown from over two interview time points. Participants (*n* = 8) were recruited from chILD patient organizations and online communities. Interview one focused on the period between January 2020 to July 2020, gaining personal insight into respondent's experience of lockdowns, which included questions on support systems and media coverage of COVID‐19. The second interview enquired how respondents managed further UK lockdowns between September 2020 and May 2021. The main themes were uncertainty and adaptation. Respondents described how they navigated the UK lockdowns and undertook various risk management strategies for pandemic isolation. Once these were established, routine and positive family bonding was reported, along with a reluctant acceptance of the COVID‐19 virus and continued shielding. As new COVID‐19 information emerged, risk management strategies changed or remained for some respondents, bringing a feeling of living with COVID‐19 as a “new normal”. (Understanding the unique insights people with rare diseases such as chILD face during a global pandemic adds to policy and healthcare literature. Recommendations include further study of caregiver traits and resilience, essential facets of positive pandemic adaptation.

## 1 Introduction

Coronavirus‐19 (COVID‐19) is a novel severe viral acute respiratory syndrome [1]. In the early stages of the COVID‐19 pandemic, the health risks for those affected with chronic respiratory conditions were unknown. Early systematic review and meta‐analysis on the clinical manifestations of COVID‐19 revealed that children generally developed a milder infection than adults [2, 3]. In April 2020, the Royal College of Paediatrics and Child Health (RCPCH) released guidance on shielding for children and young people with chronic conditions [4]. Shielding entailed staying home and avoiding face‐to‐face contact with individuals outside their household, except for essential medical purposes. Deemed as “Clinically Extremely Vulnerable” (CEV), a wide range of pediatric chronic diseases were included to protect children and young adults (CYAs) against contracting COVID‐19.

The RCPCH identified Interstitial Lung Disease in childhood (chILD) as one COVID‐19 at‐risk group. ChILD is a group of rare, chronic respiratory diseases where viral infections can trigger pulmonary exacerbations or have the potential to lead to accelerated lung damage [5]. Due to the rarity of chILDs, clinical outcome literature on COVID‐19 and chILD is sparse [6, 7]; however, early emergent literature on COVID‐19 enabled clinicians to better understand clinical COVID disease and thus allow changes to subsequent RCPCH guidance [5]. Despite the emergency clinical response of chILD, the emotional and social aspects of the COVID‐19 pandemic characteristics were overlooked. Our study sought to address this gap by exploring the psychosocial experiences of the COVID‐19 pandemic of families living with chILD. Using a qualitative longitudinal research (QLR) design to capture the temporal change in health situations [8, 9], this study examined the lived experiences of COVID‐19 UK‐based lockdowns for those affected with chILD.

To our knowledge, this is the first qualitative study to focus on the lived experience of COVID and chILD. Our findings seek to inform health policy and research practice by learning about the needs of this complex and rare to ultra‐rare group of conditions in a pandemic setting.

## 2 Methods

**Figure 1:**
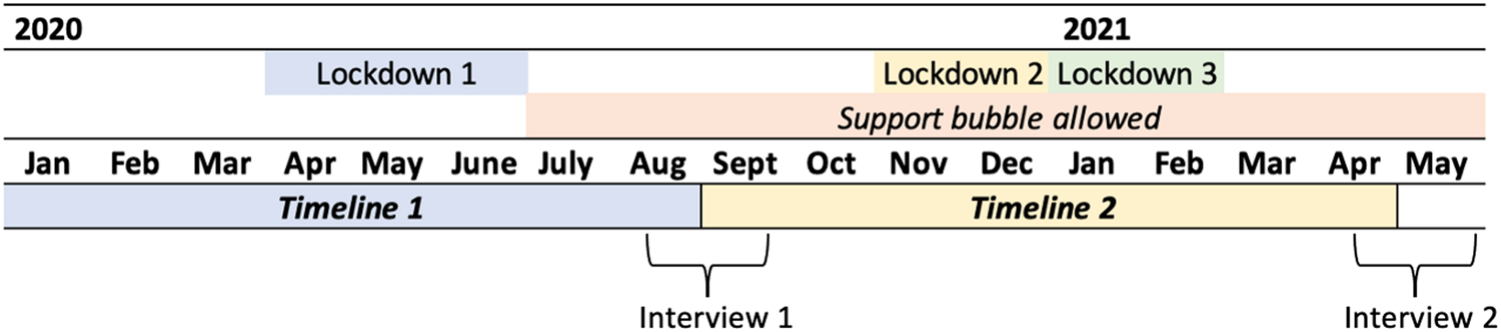
Timeline of interviews in conjunction with UK government COVID‐19 lockdowns.

During the UK pandemic, interviews were conducted at two critical time points (T1 and T2) (Figure 1). T1 covered the pre‐pandemic to the end of the first UK COVID‐19 wave (January 2020 to June 2020), with the interviews undertaken in August and September 2020. T2 explored a timeframe between September 2020 and May 2021, which covered two further UK lockdown waves (November and December 2020 and January to February 2021).

### 2.1 Participants

Participants were recruited from chILD‐related patient organizations and online community support groups. We recruited eight parents with children affected by chILD. Table 1 presents participant details: the child's age and gender, the child's ILD diagnosis, and whether they participated in the second (T2) interview.

**Table 1:** Respondent information.

Participant	Age of young person	Gender	Diagnosis	T2 interview
Parent 1 (P1)	8	Female	Bronchiolitis Obliterans	No
Parent 2 (P2)	11	Male	Surfactant protein deficiency *ABCA3*	Yes
Parent 3 (P3)	8	Female	Neuroendocrine cell hyperplasia of Infancy	Yes
Parent 4 (P4)	5	Male	Bronchiolitis Obliterans	Yes
Parent 5 (P5)	10	Female	Bronchiolitis Obliterans	Yes
Parent 6 (P6)	11	Female	Bronchiolitis Obliterans	No
Parent 7 (P7)	9	Male	Pulmonary Interstitial Glycogenosis	Yes
Parent 8 (P8)	4	Male	Neuroendocrine cell hyperplasia of Infancy	Yes

The study received ethical approval from The University of Liverpool Institute of Population Health Research Ethics Committee (ref: 7861). An amendment was accepted to conduct a follow‐up interview. All participants provided informed consent over the two interview time points.

### 2.2 The Interviews

#### 2.2.1 Timepoint 1 (T1)

Eight interviews were conducted between August and September 2020 using Zoom or Microsoft Teams. Interviews lasted between 27 and 52 min. Before consenting to the interview, respondents were sent a participant information sheet assuring that their responses would be treated confidentially and anonymized. The informed consent process was an online signature before conducting the interview.

Semi‐structured interviews were used for the interview schedule. Interviews were retrospective and chronological, allowing for reflection and exploration from January 2020 to July 2020 (Figure 1). Example questions included “*What are your experiences of support during the pandemic*?” and “*What do you think about the way people are talking about the virus?*”

#### 2.2.2 Timepoint 2 (T2)

All original respondents were contacted in April 2021 requesting a follow‐up interview. Six respondents agreed, and interviews were conducted between April and May 2021. Five interviews were conducted on Zoom. One interview was conducted via telephone, and notes were taken and checked by the respondent for accuracy. Interviews lasted between 14 and 23 min. Before consenting to the interview, respondents were resent the participant information sheet and reconsented via an online signature before conducting the interview.

One central question for this follow‐up interview was, “H*ow have you and your family been regarding COVID since we last spoke?*” This facilitated an individualised approach to assessing change between time points in line with healthcare and policy in QLR methodology [10, 11].

### 2.3 Analysis

All interviews were audio‐recorded and transcribed verbatim. All identifying features within the interviews, such as names, locations, and hospitals, were removed from the transcripts to ensure confidentiality. Age and chILD diagnosis were retained. Transcripts were analyzed using the constructivist grounded theory method [12]. NVivo 12 software was utilized for line‐by‐line coding, facilitating the exploration of emerging themes in the data. Quotes were carefully selected to preserve the participants' voices and personal experiences without introducing researcher bias. Each transcript was reflexively compared to identify commonalities, categories, and overarching themes. The coding, category building, and text analysis were conducted iteratively, leading to the development of central themes.

## 3 Results

Using a chronological narrative approach, the overarching themes of Uncertainty and Adaptation were developed from the T1 and T2 interviews. Selected quotes and additional information have been provided in the Supporting Information.

### 3.1 Uncertainty

The theme of uncertainty relates to the anticipation of the pandemic and how respondents planned and navigated through the early pandemic phase. All participants discussed fear of their child contracting COVID‐19 and their motivations to predominately protect the child from the unknown health implications of the novel virus. Respondents described how they anticipated the impact of the virus before national lockdowns and undertook goal‐directed behaviors to mitigate the risk of COVID‐19 infection. Despite respondents building access to resources, knowledge, and support, several areas prompted continued uncertainty: the media portrayal of COVID‐19 and the unknown of COVID‐19 transmission risk. This continued uncertainty developed into a significant stress and an anxiety‐based response.

All participants discussed motivations for protecting their child diagnosed with chILD from COVID‐19 infection. Due to the chILD historical and medical context, respondents described avoidance of contracting COVID‐19 as being their main focus and priority.

For many participants, there was a sense of anticipation as they discussed news sources from mid‐January 2020, stating it was “*straight on our radar*” (P4) and “*watching it carefully*” (P5), with a feeling of it being like “*a ticking bomb*” (P2). Thus, six respondents anticipated the pandemic and engaged in pre‐pandemic planning actions. These actions included stockpiling food and essential supplies, advanced booking of online shopping delivery slots, purchasing extracurricular books and resources for the children, and, with some respondents, organising working from home.

All respondents had removed their child from school before the UK official lockdown date (26 March 2020). When shielding was introduced, this was a relief to many of the parents.

Once in their home lockdown, many respondents reported obstacles to basic needs during their primary concern. Lack of food availability, such as “*fresh food*” (P2, P5) and toilet roll, was reported in the interviews. Respondents reported the need to use various support systems around this early lockdown phase. Their primary support was from family, neighbours, and friends who would help with food shopping. Early in the pandemic, respondents received shielding letters confirming their child's CEV status. Respondents felt “*comforted*” (P7) as this allowed respondents to receive priority grocery slots for online supermarket shopping or access to their local council for food and resource support.

Respondents used the news and online media sources to gather information on COVID. However, news and information were a “*mixed reality*” (P8) as respondents found information online and in the mainstream media inconsistent. This left many respondents feeling “*obsessed*” (P3), using different media forms and outlets to gather as much information as possible and finding little relief.

Due to a lack of information regarding the COVID‐19 virus, respondents discussed strategies for managing risk and decontamination, thus trying to mitigate COVID‐19 risk (Supporting Information S1). Using the information made available to them through public health sources and news outlets, along with their own experience managing their child's condition, they built their strategies for managing COVID‐19 risk. Early public health guidance on COVID‐19 prevention emphasized handwashing and the use of hand sanitizers along with mask‐wearing; many respondents described “*doing this anyway*” (P5) and having an awareness of “*touchpoints*” (P7).

Respondents described setting up “*cleaning stations*” (P1) by using porches or unused areas of their home; items or deliveries would be cleaned down with spray cleanser or alcohol gel and left for up to 3 days. Increased use of face masks, handwashing, hand sanitizer, the removal of clothes, and body washing after returning to the home were decontamination strategies used to minimise COVID risk. These strategies were applied to all members of the household.

During those times of the pandemic, uncertainty was a common feeling, and all respondents used anxiety‐based phrases to describe feelings of paralysis, helplessness, and fear (Supporting Information S1). Along with “*intense*” (P8), “*hypervigilance*” (P4) and being “*on edge*” (P1), some respondents also exhibited a “*hope for the best and prepare for the worst*” (P3) outlook.

### 3.2 Adaptation

Despite the uncertainty during the pandemic, respondents adapted to their new home life and described how they navigated the changed healthcare system and the outside world. Alongside the strategies employed to mitigate COVID‐19 risk, this acceptance allowed respondents to adjust to the unfolding pandemic. Due to the interplay between acceptance and adjustment within the interviews, this is presented as one theme. The process of adapting was present both in the T1 and in the T2 interviews and the formation of a “new normal” with living with COVID‐19.

Within T1, the first lockdown easing of restrictions was applied in July 2020. This was described as “*rushed*” (P2), with respondents feeling “*apprehensive*” (P1) and “*hesitant*” (P3). Alongside this reluctance was also an acceptance that respondents would “*just plod on*” (P5) and were “*going to have to accept that we're going back to normal*” (P8). Along with this acceptance was planning the scenario of the child contracting COVID‐19. Despite expressing continued fears of COVID‐19 infection, respondents expressed confidence in their skills and knowledge in keeping their children safe.

In June 2020, the UK Government implemented the “bubble” to mitigate the impact of COVID‐19 lockdown loneliness and isolation. Respondents discussed parents/grandparents and carers being part of their bubble and how good it was to spend time with others again. Many discussed that this was managed through COVID testing before visits or refraining from visiting if a mild infection was reported. P8 described feeling frustrated as their parents did not understand the “bubble” system and the parents were seeing other family members along with their own. Regarding outside life, four respondents were still “*reluctant*”. The main outside activity reported was walking with friends or family, but they did not engage in other forms of socialization. Many participants stated they would limit outside contact until a vaccine was available. Despite some concerns about safety and efficacy, all respondents were favorable to taking the vaccine if it allowed for a return to some normality.

Acknowledging the challenges of life in lockdown, seven of the respondents described the T1 lockdown to be a family bonding experience: a “*silver lining*” (P8). Lockdown presented an opportunity for quality family time: from hobbies such as family walks, gardening, reading, and cooking.

Over this time, most respondents reported no change in access to chILD‐related medicines. Before the pandemic, two respondents were already set up with online pharmacy delivery services (P1, P5), and during the early lockdown, all respondents were set up for a regular pharmacy delivery service. For home oxygen therapy (HOT), parents reported no oxygen provision change over this lockdown period (P2, P3, P4, P7). Respondents reported being prepared for COVID‐19 as they had access to their own purchased thermometers and oxygen saturation monitors. The most complex care support arrangement was for P7 (PIG), whose child required overnight care for tracheostomy management. P7 reported issues with consistent care provision over this period as members of the care team were clinically vulnerable themselves. P7 described the impact of poor sleep, which was not a “*workable*” long‐term solution.

Respondents reported a change of contact with their healthcare team for hospital consultations around the T1 period. Respondents understood health service pressures, and therefore, contact with the healthcare team would be limited, but they would be available if needed. Hospital appointments were canceled except for two respondents who attended telephone or video consultations with their child's respiratory consultant. One respondent noted: “*They've been so much easier than having to go to hospital*” (P4). Other respondents stated they did not need to contact their hospital during the lockdown period as the child's health was stable. Two respondents described hospital admissions over this period for their chILD condition, with one describing the experience as “*once we got there, it actually felt really safe*” (P8).

In the T2 interviews, a review of CEV status in October 2020 changed shielding for two respondents whose children were affected by NEHI. Respondents reported being “*happy and relieved* [for] *normality*” (P3) and a feeling that “*a lot of the fear is reduced*” (P8).

The UK COVID‐19 vaccination programme became available in December 2020. All respondents stated that they had received at least one COVID‐19 vaccine dose at the T2 interview. One respondent described that their child had received an exceptional circumstances review and had recently received one dose of the vaccine. It was described as a very “*emotional*” experience (P5). Despite having a minimum dose of the COVID‐19 vaccine, four respondents reported that they were still undertaking shielding and risk management strategies for COVID‐19 prevention as they still wanted to protect against COVID‐19 (P2, P4, P5, P7). Respondents described a yearning for “*some normality*” (P4) but still expressed a concern for risk. The risk management strategies included continued access to the CEV online shopping service and continual monitoring of local COVID‐19 cases for school attendance.

From a general outlook, there was a difference between those parents who were reluctant to engage with outside life and NEHI parents who no longer felt the shielding pressures. From a new‐found appreciation of life and there being “*more to everything*” (P3), this contrasted with an uncertain and questioning response of the future from the other four respondents with the proposed up‐and‐coming “Freedom Day” (19 July 2021).

There was a continued feeling that despite changes to a nationwide COVID strategy, there was still lingering uncertainty. Not only on the impact of the pandemic on the respondents and their children but also the impact on broader society and their own need to develop a new normal.

## 4 Discussion

This study provides insight into the experience of COVID‐19 lockdowns for UK‐based parents with a child with chILD. With two interviews spanning a timeframe between January 2020 and May 2021, our findings present a chronological process of uncertainty to adaptation: from early anticipation of the COVID‐19 pandemic to the fears and uncertainties experienced and what strategies they undertook to adapt to UK COVID‐19 lockdown period.

Due to the unknown health consequences on their child's chronic respiratory condition, chILD, respondents reported significant anxiety early in the pandemic when describing the importance of protecting their child from COVID‐19. Early COVID‐19 literature into the insights of adults with chronic lung conditions supports a similar theme of uncertainty [13]. To manage this, respondents developed several COVID‐19 risk management tasks such as stockpiling to avoid scarcity [14], removing the child from school before the national lockdown, establishing their support networks, and developing their own cleaning/decontamination procedures.

In comparison to other health conditions, anxiety related to the COVID‐19 pandemic appears variable in the literature. While children with chronic health conditions were generally not reported to experience significantly higher distress than healthy controls [15], those with chronic respiratory diseases, such as chronic lung disease (CLD) and cystic fibrosis (CF), showed notable psychological impacts. Children with CLD reported higher anxiety levels than controls, with parents frequently adopting a problem‐solving coping approach [16] to manage pandemic‐related challenges. Similarly, adolescents with CF and their parents expressed heightened anxiety, fear, and feelings of being overwhelmed, emphasizing the need for tailored resources to support them through the pandemic. This pervasive fear and uncertainty regarding the immediate and long‐term implications of novel respiratory viruses and the development of a risk‐management approach among families align closely with our interview findings. Adult respiratory conditions reported utilizing similar task‐related problem‐solving strategies when navigating the pandemic and increased anxiety [17, 18]. Since this research was conducted in an emergency, a quality assessment of the literature focusing on those affected with a chronic health condition would help understand the impact on mental health and coping strategies during the COVID‐19 pandemic.

Despite feelings of uncertainty and anxiety, the respondents' risk management strategies laid the foundations for experience building, such as establishing a routine and navigating the new healthcare landscape. Changes in healthcare services during the pandemic were not reported to be an issue of concern for these respondents. This ran counter to other non‐UK literature where there were challenges in healthcare service provision [19, 20]. It must be noted that due to the complexity of chILD care, parents have become experts in caring for their own child [21]. In addition to the confidence in caring for the child with ILD, positive family bonding allowed respondents to create an environment that allowed for adaptation: “*a silver lining*” [22]. Where skill‐building and family bonding have been reported to be positively associated with motivation, self‐efficacy, and health status in other COVID‐related qualitative studies [23, 24], our findings suggest that the role of self‐efficacy did help ameliorate uncertainty and act as a supporting factor for successful adaptation for these respondents [25]. The role of self‐efficacy and resilience is an interesting factor in how parents of children with complex chronic health conditions such as chILD can plan and adapt in challenging and uncertain situations. Since there is no research within this area, this is a recommended further study area for chILD families and other rare disease populations.

### 4.1 Strengths and Limitations

One key strength of this study is the novel qualitative longitudinal insight into the unique experiences and challenges of parents with children affected with chILD during the UK lockdowns. Collecting experiences and understanding aspects of care builds patient engagement with rare disease communities and has the ability to collect novel insights specific to this cohort.

This study's limitations include chILD community recruitment. Online recruitment may present issues in sample representation, such as the exclusion of families with access to digital services and the risk of potential bias due to community activity. Due to the CGT methodology used in planning the study and researcher triangulation, this acknowledges and processes any possible bias. The study was only available to English speakers, and including non‐English speaking minority groups within the UK would have improved the study generalizability of the pandemic response. Another limitation includes the lack of control group data and potential cohort sample size. Data saturation is a component of qualitative research to ensure content validity; however, this is situation‐dependent. Since ChILD is comprised of rare to ultra‐rare conditions with variable prognoses, the representation of rare disease voices in psychosocial research should be actively encouraged by using ethical and pragmatic means.

## 5 Conclusion

This study's retrospective and chronological structure enabled participants to reflect on the UK COVID‐19 pandemic between January 2020 and May 2021. One of the key findings consistent across the two interviews is how pandemic uncertainty evolved into adaptation. Since uncertainty about health and the future is common among individuals with chronic and rare diseases, the pandemic aggravated these uncertainties through fear of contracting COVID‐19 infection. Once settled into a routine with resources and an acceptable level of controlled safety, many respondents discussed lockdown as a positive experience for family bonding. As the pandemic unfolded and more health information became available along with the introduction of vaccinations, respondents developed a reluctant acceptance and adapted to a “new normal” of living with COVID‐19. To better support families with children affected by a complex respiratory condition, increased flexible resources, along with psychological support to address chronic condition‐specific stressors, may ensure continuity of care. Together, these approaches can help strengthen family coping mechanisms during future public health crises. These findings highlight a broader discussion on resilient traits and coping styles among parents with complex chronic health conditions, with this study adding to the literature on pandemic experiences for health policy and research.

## Author Contributions

**Carlee Gilbert:** conceptualization, writing–original draft, writing–review and editing, methodology, investigation, formal analysis, data curation, project administration, software. **Andrew Bush:** writing–review and editing, formal analysis. **Kate M. Bennett:** supervision, writing–review and editing, writing–original draft, data curation, investigation, formal analysis. **Christopher Brown:** investigation, supervision, writing–original draft, writing–review and editing.

## Supporting information

Supporting information.

## Data Availability

The data that support the findings of this study are available from the corresponding author upon reasonable request.
