# Supplementary material for: “We Could Hold Our Own Here at Home”: Longitudinal Experience of COVID‐19 Lockdowns in Parents With Children Affected With Interstitial Lung Disease
Source: Pediatr Pulmonol. 2024 Dec 18;60(1):e27446. doi: 10.1002/ppul.27446 (PMC11748111; doi:10.1002/ppul.27446)
Supplement: Supplementary file 1 — Supporting information. [file PPUL-60-0-s001.docx]

**Supplemental Data**

Uncertainty Theme

Quotes supporting the focus and priority being the avoidance of the COVID-19 virus:

“*So* *worried about “H” because she has done so well… I wouldn’t want anything to happen to send her lungs backwards*.” (P6)

“*RSV. This obliterated his lungs…. with genetic ABCA3, it was a cocktail of lung disaster. A virus did that to him once before. We didn’t even wish to think about what this virus [COVID-19] that was killing hundreds of people would do to him. He needed protecting.”* (P2*)*

Selected quote of relief for lockdown and shielding:

“*10 days before and it just seemed that we were hearing these stories, but nothing was quite happening. ... And I just thought, I can't risk it. You can only make the decision once. And if we get that wrong and it is that serious for her to get it then, so we just said, ‘right, that's it - she's coming home*”.” (P3)

Selected quote of use of media and consuming information early pandemic:

“*I was just constantly watching … reading the news, just not knowing what to do. I was just awful in the beginning. It was really, really stressful*.” (P4)

Table 1: *Strategic tasks respondents undertook during the pandemic to manage uncertainty*

| Strategy | Action |
| --- | --- |
| Pre-pandemic planning | Removing children from school |
|  | Stock piling of food |
|  | Collecting resources |
|  | Setting up online services |
| Establishing avenues of support | Health and Government support |
|  | Personal support systems |
| COVID-19 in the media | Gathering accurate information |
| Mitigating COVID risk | Working arrangements |
|  | Setting up cleaning stations |
|  | Vaccine |

Table 2: *Respondents describing their COVID experience related to uncertainty*

| “*It’s a strange time… Everything kind of stopped*” (P1) |
| --- |
| “*The inaction was frustrating as we… could see everything unfold.*” (P2) |
| “J*ust bizarre*” (P3) |
| “*Not knowing what to do*” (P4) |
| “J*ust go cry in the shower because it's just such a surreal feeling*” (P5) |
| “N*one of us knew how this was going to be*” (P6) |
| “*Just scary*” (P7) |
| “*Really, really uncertain*” (P8) |

Adaptation theme

Selected quote on confidence in keeping their child safe and isolated from COVID-19:

“*We're quite fortunate in that he's got a ventilator and I know what I'm doing with it. It's quite odd being in charge of people's breathing because I know what to do to artificially make it be all right for a bit. So, I'd like to think that if he got it [COVID-19], we could hold our own here at home*.” (P7).

Selected quote for vaccine acceptance:

“*we're never going to get out of this without a vaccine*” (P1)

Selected quotes on feelings for the future of living with COVID-19:

“*That is sad in a way. Another year of our lives gone. A year of my children’s lives gone. And what we have left is what we have to pick up the pieces with. Where do we go from here? Because it doesn’t feel over. Not at all*.” (P2)

“*I’d say we are living like we just have to be careful and hope everybody's doing the same… Or something like that. <laughter>*” (P7)
